# Spectral Unmixing Imaging for Differentiating Brown Adipose Tissue Mass and Its Activation

**DOI:** 10.1155/2018/6134186

**Published:** 2018-01-04

**Authors:** Jing Yang, Jian Yang, Chongzhao Ran

**Affiliations:** ^1^Molecular Imaging Laboratory, MGH/MIT/HMS Athinoula A. Martinos Center for Biomedical Imaging, Department of Radiology, Massachusetts General Hospital/Harvard Medical School, Room 2301, Building 149, Charlestown, Boston, MA 02129, USA; ^2^School of Pharmacy, Soochow University, Suzhou 215006, China; ^3^Center for Drug Discovery, School of Pharmacy, China Pharmaceutical University, Nanjing 210009, China

## Abstract

Recent large-scale clinical analysis indicates that brown adipose tissue (BAT) mass levels inversely correlate with body-mass index (BMI), suggesting that BAT is associated with metabolic disorders such as obesity and diabetes. PET imaging with 18F-FDG is the most commonly used method for visualizing BAT. However, this method is not able to differentiate between BAT mass and BAT activation. This task, in fact, presents a tremendous challenge with no currently existing methods to separate BAT mass and BAT activation. Our previous results indicated that BAT could be successfully imaged in mice with near infrared fluorescent (NIRF) curcumin analogues. However, the results from conventional NIRF imaging could not reflect what portion of the NIRF signal from BAT activation contributed to the signal observed. To solve this problem, we used spectral unmixing to separate/unmix NIRF signal from oil droplets in BAT, which represents its mass and NIRF signal from blood, which represents BAT activation. In this report, results from our proof-of-concept investigation demonstrated that spectral unmixing could be used to separate NIRF signal from BAT mass and BAT activation.

## 1. Introduction

Brown adipose tissue (BAT) has been considered as “good fat,” due to its function of dissipating large amounts of chemical/food energy as heat to maintain the energy balance of the whole body [1–3]. Investigations of BAT have been ongoing for decades, particularly using animals. Reportedly, BAT has been assumed to have no physiologic relevance in adult humans, even though it is highly abundant in embryonic and early postnatal stages. However, this dogmatic opinion has been overturned by large clinical studies. In 2009, Cypess et al. reported that, by analyzing 3640 PET-CT images of 1,972 patients, BMI (body-mass index) inversely correlated with the amount of BAT, strongly suggesting that BAT is an important target in obesity and diabetes [4]. The existence of BAT in adults has been strongly endorsed by other important investigations as well [5–11]. Moreover, since 2009 numerous groundbreaking studies strongly support the significance and potential benefits of BAT [12–33]. Characteristically, BAT contains a large number of mitochondria, abundant uncoupling protein-1 (UCP-1) expression, numerous small oil droplets in a single cell, and significant vascularization of BAT tissue [4, 34–37]. The above characteristics strongly imply that BAT plays an important role in metabolism and energy expenditure; therefore BAT is a potential target for diabetes and obesity therapy.

The assumption that BAT is “nonexistent” in adults is partially due to the lack of proper imaging methods to “see” the small BAT depots* in vivo*, as only 3%–8% of adult patients' BAT depots could be clearly visualized with 18F-FDG if no cold or drug stimulation is applied [38–40]. However, under stimulated conditions, PET-FDG imaging has shown that BAT is still present in 95% health adults in the upper chest, neck, and other locations [4, 6, 8, 34, 35]. This remarkably large difference between unstimulated and stimulated conditions strongly indicates that PET-FDG imaging only reflects the activation of BAT, but not BAT mass. Therefore, imaging probes that can consistently report BAT mass are highly desirable.

Accurately reporting BAT mass is a tremendous challenge for imaging scientists, due to the fact that BAT mass and BAT activation are intertwined under various conditions. It is obvious that there is no absolute “resting” status of BAT, and BAT activation cannot be “zero” for a living subject. Therefore, dissection of BAT mass and BAT activation is a remarkable challenge. However, most of the current imaging methods often reflect the summed signal from BAT mass and activation. Although PET-FDG imaging has significantly contributed to the “rediscovery” of BAT in adults, it primarily reflects BAT activation, but not BAT mass [41]. Similarly, most of other reported imaging methods are also BAT activation dependent [24, 41–51]. Our group has recently reported that near infrared fluorescence (NIRF) probe CRANAD-*X* (*X* = -2, -3, and -29) could be used for BAT mass imaging [52], and Cerenkov luminescence imaging with ^18^F-FDG could be applied to image BAT in mice [53]. Via conventional NIRF imaging with CRANAD-29, BAT mass change in a streptozotocin-induced diabetic mouse model and BAT activation under cold exposure could be reported. In addition, the same method could be used to monitor “browning” of WAT that was induced by *β*3-adrenoceptor agonist CL316,243 [52]. However, conventional NIRF imaging is not capable of dissecting the signal from BAT mass and from BAT activation.

To the best of our knowledge, there is no available imaging method for differentiating BAT mass and activation. The key to this challenge is to dissect BAT mass measurement from BAT physiology status (activated or suppressed). It is well known that BAT is highly vascularized, and activation of BAT is tightly closely associated with a significant increase of blood flow. Therefore, the change of blood flow has been considered to be a biomarker for BAT activation [54–56]. For a hydrophobic NIRF probe, its emission spectra are highly dependent on its environments [57]. In a hydrophobic environment such as in oil droplet of BAT mass, its emission spectra would be significantly blue-shifted [57]. Therefore, for the same NIRF probe, there will be apparent difference between the spectra from oil droplets of BAT and the spectra from blood flow. Previously, we have successfully utilized spectral unmixing technique to differentiate bound and free probe in the case of in vivo amyloid beta detection [58]. In this report, we demonstrated that spectral unmixing could be used to dissect NIRF signal from BAT mass and NIRF signal from blood flow [59]. With this technique, it is feasible to accurately report BAT mass and BAT activation/physiological status.

## 2. Methods and Materials

The reagents used for the synthesis were purchased from Aldrich and used without further purification. CRANAD-29 was synthesized according to our previously reported procedures [52]. All animal experimental procedures were approved by the Institutional Animal Care and Use Committee (IACUC) at Massachusetts General Hospital and carried out in accordance with the approved guidelines.* In vivo* NIRF imaging was performed using the IVIS® Spectrum animal imaging system (Caliper Life Sciences, Perkin Elmer, Hopkinton, MA), and data analysis was conducted using Living Image® 4.2.1 software. Mice were anesthetized with isoflurane balanced with oxygen during image acquisition (less than 5 minutes for each imaging session).

### 2.1. Ex Vivo Spectral Unmixing with Dissected BAT and Blood

A two-month-old balb/c mouse was injected intravenously with 100 *μ*L CRANAD-29 (0.4 mg/kg, 15% DMSO, 15% Cremophor EL, and 70% PBS pH 7.4). The mouse was sacrificed at 4 hours after the injection. BAT was dissected and 0.1 mL blood was collected. Sequence images were acquired with the following parameters: Ex/Em pairs: 605/660 nm, 640/680 nm, 640/700, 640/720 nm, 675/740 nm, 675/760 nm, and 675/780 nm. Exposure time is auto, FOV = B. Spectral unmixing was performed with Living Image® 4.2.1 software, and manual unmixing method was selected. The generated spectra for autofluorescence, BAT, and blood were saved as a spectral library for CRANAD-29.

### 2.2. In Vivo Spectral Unmixing of CRANAD-29 in Mice

A two-month-old balb/c mouse was injected intravenously with 100 *μ*L CRANAD-29 (0.4 mg/kg, 15% DMSO, 15% Cremophor EL, and 70% PBS pH 7.4) in a 25°C room. Sequence images were captured at 4 hours after CRANAD-29 injection with the following parameters: Ex/Em pairs: 605/660 nm, 640/680 nm, 640/700, 640/720 nm, 675/740 nm, 675/760 nm, and 675/780 nm. Exposure time is auto, FOV = D. Spectral unmixing was performed with Living Image® 4.2.1 software, and Library Unmixing Method was selected.

### 2.3. In Vivo Spectral Unmixing of CRANAD-29 in Mice under Short Cold Exposure

Two-month-old balb/c mice (*n* = 5) were placed in a 4°C cold room for 2 hours before intravenous injection of CRANAD-29. After CRANAD-29 was totally washed out (about 10 days because of the slow clearance of CRANAD-29), the same group of mice were used as the control group (*n* = 5) and were placed in a 25°C room. Sequence images were acquired at 4 hours after probe injection with the same parameters as above in vivo imaging. For the cold exposure group, the mice were maintained at 4°C for 4 hours after probe injection. Spectral unmixing was performed with Living Image® 4.2.1 software, and Library Unmixing Method was selected. ROIs were manually drawn around the BAT area.

## 3. Results and Discussions

In our previous report, with conventional NIRF imaging, we demonstrated that CRANAD-29 had significant selectivity for BAT over WAT and could be used to monitor BAT activation and BAT mass changes [52]. For a NIRF probe, its residing environments have significant impact on its fluorescence properties, including intensity, emission spectrum, and lifetime. We hypothesized that the emission spectra of the same NIRF probe were different from oil droplets in BAT mass and from blood flow, due to their different residing environments, and the spectral difference could be used for spectral unmixing.

**Figure 1:**
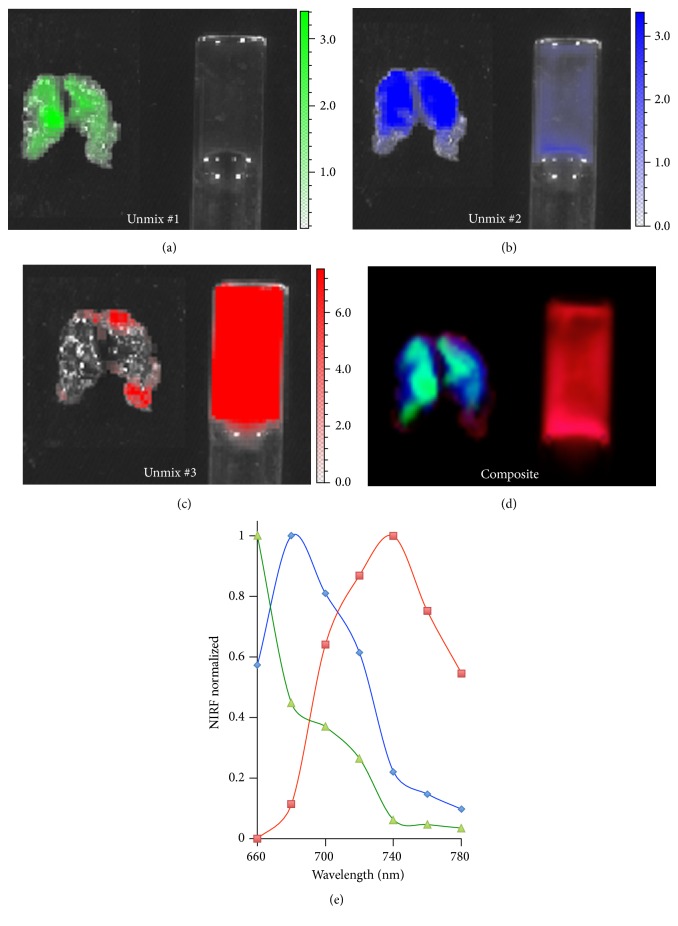
Spectral unmixing with CRANAD-29 for ex vivo BAT and blood. (a) Unmixed #1 represents autofluorescence signal and is corresponding to green line spectrum in (e). (b) Unmixed #1 represents NIRF signal from BAT mass and is corresponding to blue line spectrum in (e). (c) Unmixed #2 is for NIRF from blood and red line spectrum in (e). (d) Merged image of unmixed #1, #2, and #3. (e) Ex vivo unmixed spectra for autofluorescence (green), BAT mass (blue), and blood flow (red).

To validate our hypothesis, we first conducted spectral unmixing imaging with ex vivo BAT tissue and blood from a mouse injected with CRANAD-29. Sequence images were acquired with seven Ex/Em pairs, and spectral unmixing was conducted with Living Image® 4.2.1 software. As expected, we were able to differentiate BAT and blood, as evidenced by the well-separated images (Figures 1(a)–1(d)) and spectra from BAT and blood (Figure 1(e)). The spectra generated from this ex vivo unmixing were saved as a spectral library of CRANAD-29, which can be used for in vivo unmixing investigation.

**Figure 2:**
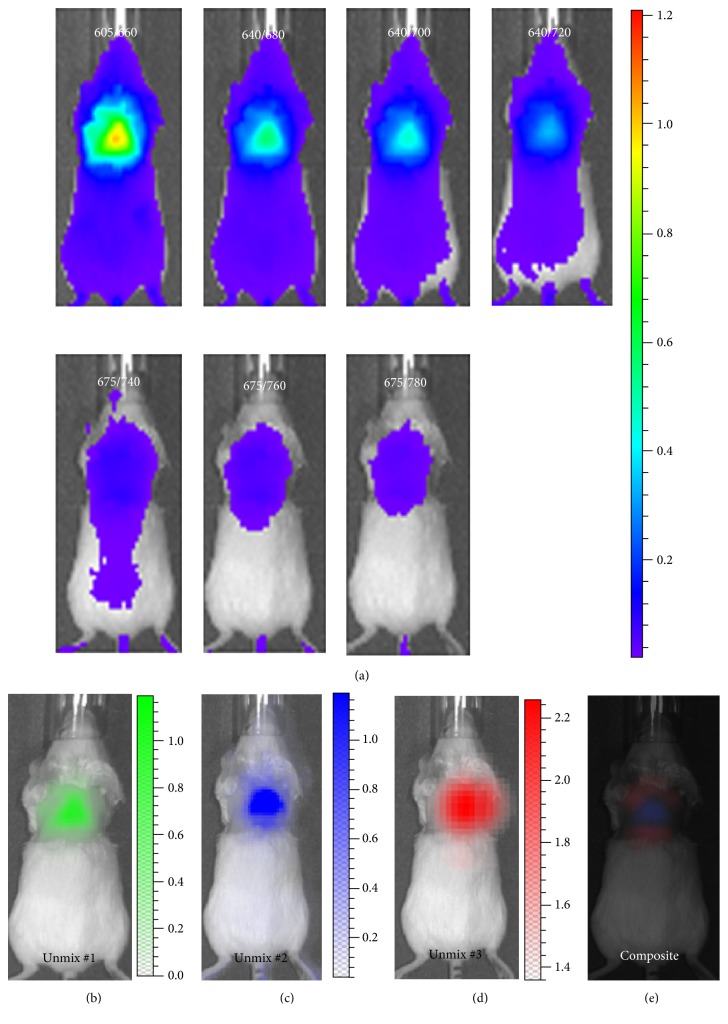
Spectral unmixing with CRANAD-29 for in vivo imaging. (a) Raw images of CRANAD-29 before spectral unmixing. (b) Unmixed autofluorescence signal. (c) Unmixed NIRF signal from BAT mass. (d) Unmixed NIRF signal from blood flow. (e) Merged image of unmixed #2 and #3. Note: for clarity, unmixed #1 was not merged.

To further validate the feasibility of spectral unmixing for in vivo studies, we acquired sequence images with the same parameters as the above ex vivo experiment with a mouse that was injected CRANAD-29. We used the spectral library of CRANAD-29 to conduct the spectral unmixing. As shown in Figure 2, the autofluorescence, signal of BAT, and blood stream could be well-separated, suggesting that the spectral unmixing is feasible for in vivo imaging.

**Figure 3:**
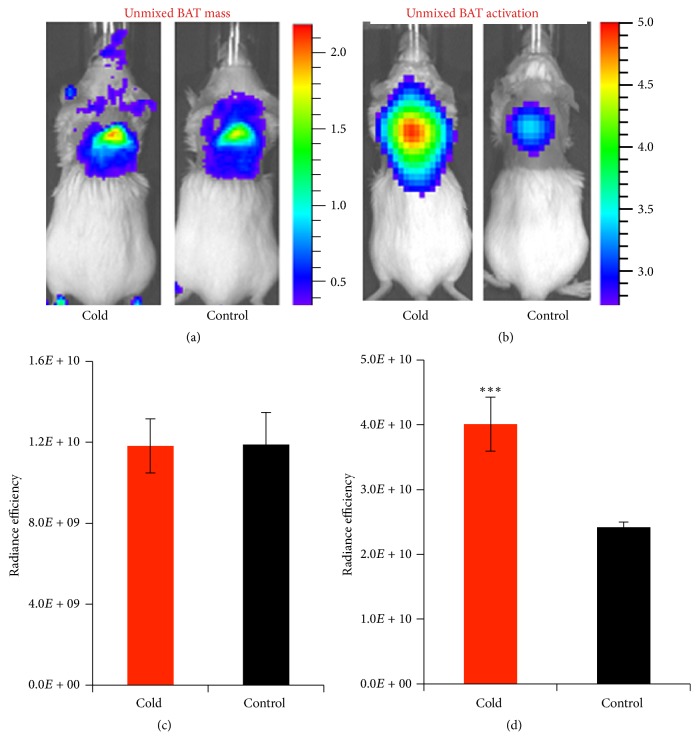
Spectral unmixing with CRANAD-29 for in vivo imaging under cold treatment. (a) Unmixed NIRF signal from BAT mass under cold treatment and control condition. (b) Unmixed NIRF signal from blood flow reflecting BAT activation. (c-d) Quantitative analysis of unmixed NIRF signal from BAT mass (c) and blood flow (d) under cold treatment and the control condition. ^*∗∗∗*^*p* < 0.005.

To investigate spectral unmixing which could be used to dissect the signals from BAT mass and BAT activation, we conducted proof-of-concept experiment with the same group of mice with and without short cold exposure. The group of mice (*n* = 5) were treated with a short cold exposure (2 hours) and injected with CRANAD-29. After 4 hours of the injection. Sequence images were captured with the same parameters as above. After CRANAD-29 totally washing out, the same mice without the cold treatment were imaged again with the probe. We compared the unmixed NIRF signals from BAT and blood flow under cold treatment and without cold exposure. Obviously, with such a short cold exposure, the BAT mass would not change, but the blood flow was expected to significantly increase under the cold treatment. Indeed, we found that there was no significant NIRF signal difference from BAT mass (Figures 3(a) and 3(c), *p* = 0.975), but an apparent increase of NIRF signal from blood flow from the cold exposure condition, and the increase was about 1.66-fold (Figures 3(b) and 3(d), *p* = 0.005). These results indicated that our method was reliable. Taken together, the above in vitro and in vivo data strongly indicated that spectral unmixing could be used to separate NIRF signal from BAT mass and BAT activation.

## 4. Conclusion

In this report, we developed a spectral unmixing method that could be, for the first time, to differentiate BAT mass and BAT activation. We believe that our method has the feasibility to reliably report BAT mass changes under different genetic manipulation and drug treatment in preclinical studies. Our cost-efficient NIRF imaging has a potential impact on preclinical animal studies and will greatly assist drug discovery and basic research related to BAT.
